# Clinical Utility and the Yield of Single Nucleotide Polymorphism Array in Prenatal Diagnosis of Fetal Central Nervous System Abnormalities

**DOI:** 10.3389/fmolb.2021.666115

**Published:** 2021-05-18

**Authors:** Meiying Cai, Hailong Huang, Liangpu Xu, Na Lin

**Affiliations:** Department of the Prenatal Diagnosis Center, Fujian Maternity and Child Health Hospital, Affiliated Hospital of Fujian Medical University, Fujian Key Laboratory for Prenatal Diagnosis and Birth Defect, Fuzhou, China

**Keywords:** central nervous system, copy number variations, obstetrics, SNP-array, prenatal assessment

## Abstract

Applying single nucleotide polymorphism (SNP) array to identify the etiology of fetal central nervous system (CNS) abnormality, and exploring its association with chromosomal abnormalities, copy number variations, and obstetrical outcome. 535 fetuses with CNS abnormalities were analyzed using karyotype analysis and SNP array. Among the 535 fetuses with CNS abnormalities, chromosomal abnormalities were detected in 36 (6.7%) of the fetuses, which were consistent with karyotype analysis. Further, additional 41 fetuses with abnormal copy number variations (CNVs) were detected using SNP array (the detection rate of additional abnormal CNVs was 7.7%). The rate of chromosomal abnormalities, but not that of pathogenic CNVs in CNS abnormalities with other ultrasound abnormalities was significantly higher than that in isolated CNS abnormalities. The rates of chromosomal abnormalities and pathogenic CNVs in fetuses with spine malformation (50%), encephalocele (50%), subependymal cyst (20%), and microcephaly (16.7%) were higher than those with other isolated CNS abnormalities. The pregnancies for 36 cases with chromosomal abnormalities, 18 cases with pathogenic CNVs, and three cases with VUS CNVs were terminated. SNP array should be used in the prenatal diagnosis of fetuses with CNS abnormalities, which can enable better prenatal assessment and genetic counseling, and affect obstetrical outcomes.

## Introduction

Fetal central nervous system (CNS) abnormality is one of the most common fetal congenital malformations, and its incidence rate is about 1% (Onkar et al., 2014). The etiology and mechanisms of fetal CNS abnormality are still unknown. A previous study has reported that about 40% of the pathogenic factors may be environmental and genetic (Huang et al., 2012). CNS abnormalities represent a broad clinical spectrum, including mild ventricular enlargement, posterior fossa widening, hydrocephaly, agenesis of the corpus callosum (ACC), and holoprosencephaly (Hadzagić-Catibusić et al., 2008). With the development of prenatal ultrasound, most fetal CNS abnormalities are found in prenatal ultrasonic examination. Fetuses with abnormal ultrasound are usually recommended to undergo invasive prenatal diagnosis, in order to determine whether they have genetic anomalies, to identify possible etiology, and assess the prognosis of the fetus.

The traditional karyotype analysis is the gold standard for the diagnosis of chromosomal abnormalities; however, it is difficult to determine the chromosomal microdeletions and microduplications using karyotype analysis, and it takes a long time for cell culture. Chromosome microarray analysis (CMA) is increasingly being used for genetic diagnosis in the medical field, such as for the diagnosis of autism, intellectual disability, developmental delay, miscarriage, or stillbirth, due to the high resolution that it offers (Cukier et al., 2014; Reddy et al., 2012; Sahoo et al., 2017). The American Guidelines for Obstetricians and Gynecologists recommend to replace traditional karyotype analysis with CMA when ultrasound detects one or more large structural abnormalities in the fetus; however this has not been extended to clinical practice (Hanson et al., 2015; Committee Opinion, 2016). CMA can be divided into two types: microarray comparative genomic hybridization and single nucleotide polymorphism (SNP) array, both of which can detect the chromosomal microdeletions and microduplications. SNP array can detect not only copy number variations (CNVs), but also uniparental disomy and chimera.

Through prenatal ultrasound screening, genetic analysis and clinical consultation, the prognosis of fetuses with CNS can be evaluated. Fetuses with severe malformations and chromosomal abnormalities can terminate pregnancy in time, which can effectively control the birth rate of fetuses with severe malformations. It is of great clinical significance for guiding prenatal and postnatal care, reducing birth defects and improving the quality of newborn population. In this study, we used SNP array to explore its clinical value in assessing fetal CNS abnormalities, to better conduct prenatal genetic counseling and evaluate the possible prognosis of fetuses according to the genetic etiology.

## Materials and Methods

### Clinical Data

**FIGURE 1:**
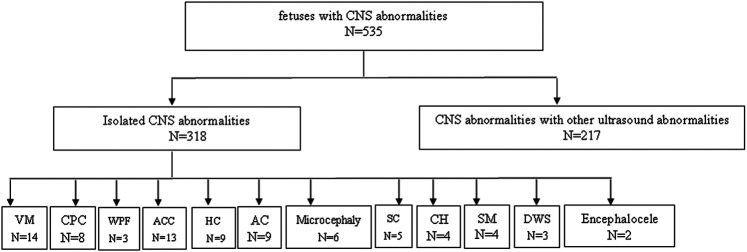
Enrollment of Study Participants, VM, ventricular widening; CPC, choroid plexes cytsts; WPF, widened posterior fossa; ACC, agenesis of the corpus callosum; AC, arachnoid cyst; SC, subependymal cyst; CH, cerebellar hypoplasia; SM, spine malformation; DWS, dandy-walker syndrome; HC, hydrocephaly.

A total of 535 pregnant women with fetal CNS abnormalities were selected from the Prenatal Diagnosis Center of the Fujian Provincial Maternal and Children Health Hospital from November 2016 to July 2020. The study were performed in accordance with the Declaration of Helsinki. All experiments were approved by the local ethics committee at the Fujian Provincial Maternal and Child Health Hospital (2014–042). All parents were wrote informed consent. The gestational age of the pregnant women ranged from 12–38 weeks, with an average of 25.4 weeks. The age of pregnant women ranged from 17–48 years, with an average age of 28.4 years. Among the 535 cases, there were 318 cases of isolated CNS abnormalities and 217 cases of CNS abnormalities with other ultrasound abnormalities. Isolated CNS abnormalities imply that ultrasound abnormalities are limited to the CNS. CNS abnormalities with other ultrasound abnormalities mean that in addition to CNS abnormalities, abnormalities involving the heart, urinary, digestive, and other systems are also present. The 318 cases of isolated CNS abnormalities included 142 cases of mild ventricular enlargement, 89 cases of choroid plexus cyst, 32 cases of widened posterior fossa, 13 cases of ACC, nine cases of hydrocephaly, nine cases of arachnoid cyst, six cases of microcephaly, five cases of subependymal cyst, four cases of cerebellar hypoplasia, four cases of spine malformation, three cases of Dandy-Walker syndrome, and two cases of encephalocele (Figure 1).

### Karyotype Analysis

According to the routine methods established by our center (Cai et al., 2020), cells from the villi, amniotic fluid, or umbilical cord blood samples of the 535 fetuses with CNS abnormalities were cultured, harvested, prepared, and G-banded. The prepared samples were collected and analyzed using the GSL-120 automatic chromosome scanning platform.

### SNP-Array

Genomic DNA amplification, hybridization, scanning, and data analysis were performed using the Affymetrix’s CytoScan 750 K chip and Illumina kit following the standard operating procedures. Results were interpretated using the following reference databases: DGV (http://projects.tcag.ca/variation), ISCA (https://www.iscaconsortium.org/), OMIM (http://www.omim.org), DECIPHER (htts://decinher.sanger.ac.uk/). According to the corresponding criteria, the results were divided into benign CNVs, CNVs with uncertain clinical significance (VUS), and pathogenic CNVs. In the case of abnormal CNVs, parental samples were also tested to determine the genetic nature of the variation and its clinical significance. Pedigree validation was suggested to all fetuses with pathogenic or probably pathogenic CNVs and VUS CNV to investigate with SNP array to evaluate a possible recurrence risk. The classification of VUS CNV will be reassessed with pedigree validation results.

### Statistical Analysis

SPSS 22.0 software was used for statistical analysis. The rates of chromosomal abnormalities and pathogenicity CNVs were compared between isolated CNS abnormalities groups and CNS abnormalities with other ultrasound abnormalities groups. The χ^2^ test was used to compare in groups and the difference was considered statistically significant when *p* < 0.05.

## Results

### Karyotype Analysis of 535 Fetal CNS Abnormalities

Among the 535 fetuses with CNS abnormalities, 36 fetuses had pathogenic chromosomal abnormalities (6.7%, 36/535), including trisomy 18 (*n* = 11), trisomy 21 (*n* = 8), trisomy 13 (*n* = 3), other chromosome number abnormalities (n = 2), chromosomal structural abnormalities (*n* = 12; 46,XY,r (p22q36), 46,XY,r (18) (q10), 46,XY,add (21) (q11.2), 46,XX,add (12) (p13.33), 46,XY,del (5) (p14.3), 46,XX,add (5) (p15.3), 46,XX,del (15) (p13), 46,XY,del (4) (q25q28), 46,XY,del (5) (q21q31), del (11) (q14q22), 46,XX,del (13) (q31q34), 46,XY,del (5) (p15p13), 46,X,add (X) (q28)). The fetuses with pathogenic chromosomal abnormalities were resulted in pregnancy termination (Table 1). The rates of chromosomal abnormalities were 4.1% (13/318) in isolated CNS abnormalities, and 10.6% (23/217) in CNS abnormalities with other ultrasound abnormalities. The rate of chromosomal abnormalities in CNS abnormalities with other ultrasound abnormalities was significantly higher than that in isolated CNS anomalies, and the difference was statistically significant (*P* = 0.003, *p* < 0.05) (Table 2).

**TABLE 1:** Pathogenic chromosomal abnormalities in fetal CNS abnormalities.

Case	Karyotype	CNS abnormalities	Extra CNS abnormalities
1	47,XX,+18	Choroid plexus cyst	−
2	47,XY,+18	Choroid plexus cyst	−
3	47,XY,+21	Choroid plexus cyst	−
4	47,XY,+21	Choroid plexus cyst	−
5	47,XX,+13	Choroid plexus cyst	−
6	47,XX,+21	Posterior fossa widened	−
7	45,X	Posterior fossa widened	−
8	47,XX,+21	Mild ventriculomegaly	−
9	46,XX,add (12) (p13.33)	Mild ventriculomegaly	−
10	46,XX,del (15) (p13)	ACC	−
11	47,XX,+21	Microcephalus	−
12	46,XY,r (p22q36)	Spine malformation	−
13	46,XY,r (18) (q10)	Subependymal cyst	−
14	47,XX,+18	Posterior fossa widened	Pyelectasis, Single umbilical artery
15	47,XY,+18	Choroid plexus cyst	CHD
16	47,XY,+18	Choroid plexus cyst	VSD, Strephenopodia
17	47,XY,+18	Choroid plexus cyst	Echogenic bowel
18	47,XY,+18	Mild ventriculomegaly	Left ventricular hyperecho
19	47,XY,+18	HPE	CHD
20	47,XX,+18	Choroid plexus cyst	CHD
21	47,XY,+18	Choroid plexus cyst	CHD
22	47,XX,+18	Choroid plexus cyst	CHD
23	47,XY,+21	Mild ventriculomegaly	Thickened nuchal translucency
24	47,XX,+21	Choroid plexus cyst	Thickened nuchal translucency, Absent nasal bone
25	47,XY,+21	Mild ventriculomegaly	Absent nasal bone
26	47,XX,+13	HPE	Lip and palate clef, CHD
27	47,XX,+13	HPE	Lip and palate clef, CHD
28	45,X [30]/46,XX [70]	Posterior fossa widened	Echogenic bowel
29	46,XY,add (21) (q11.2)	Mild ventriculomegaly	VSD
30	46,XY,del (5) (p14.3)	Cerebellar hypoplasia	Thickened nuchal translucency, right aortic arch
31	46,XX,add (5) (p15.3)	Spine malformation	Short femur
32	46,XY,del (4) (q25q28)	Mild ventriculomegaly	VSD
33	46,XY,del (5) (q21q31), del (11) (q14q22)	Choroid plexus cyst	Cystic hygroma, Echogenic bowel
34	46,XX,del (13) (q31q34)	HPE	FGR
35	46,XY,del (5) (p15p13)	Cerebellar hypoplasia	Thickened nuchal translucency
36	46,X,add(X) (q28)	Choroid plexus cyst	FGR

ACC, agenesis of the corpus callosum; CNS, central nervous system; CHD, congenital heart disease; FGR: fetal growth restriction; HPE, holoprosencephaly; P, pathogenicity; VSD: ventricular septal defect.

**TABLE 2:** The rates of chromosomal abnormalities in isolated CNS abnormalities and CNS abnormalities with other ultrasound abnormalities.

Classification	Number of fetuses	Number of chromosomal abnormalities	Trisomy 18	Trisomy 21	Trisomy 13	Other chromosome number abnormalities	Structural chromosomal abnormalities	p-values
Isolated CNS abnormalities	318	13 (4.1%)	2	5	1	1	4	−
CNS abnormalities with other ultrasound abnormalities	217	23 (10.6%)	9	3	2	1	8	−
Total	535	36 (6.7%)	11	8	3	2	12	0.003

p < 0.05 compared with isolated CNS anomalies and CNS abnormalities with other ultrasound abnormalities via χ^2^ test.

CNS, central nervous system.

### Single Nucleotide Polymorphism Array of 535 Fetal CNS Abnormalities

Among the 535 fetuses with CNS abnormalities, in addition to 36 fetuses of chromosomal abnormalities that were detected using karyotype analysis, additional 41 fetuses of abnormal CNVs were detected by SNP array. The detection rate of additional abnormal CNVs was 7.7% (41/535). Among 41 fetuses with abnormal CNVs, 18 were pathogenic CNVs, and 23 were VUS CNVs. In the 318 cases of isolated CNS abnormalities group, there were a total of 26 cases with abnormal CNVs, including eight cases with pathogenic CNVs and 18 cases with VUS CNVs. In the 217 cases of CNS abnormalities with other ultrasound abnormalities group, there were a total of 15 cases with abnormal CNVs, including 10 cases with pathogenic CNVs and five cases with VUS CNVs. The size of the CNVs detected was between 0.2 and 7.7 Mb. Among the 18 cases of pathogenic CNVs, eight cases had known microdeletion/microduplication syndrome, five cases had 16p11.2 microdeletion syndrome, two cases had 22q11.2 microdeletion syndrome, and one case had Miller-Dieker syndrome (17p13.3p13.2 microdeletion syndrome) (Table 3). Among 23 fetuses with VUS CNVs, 15 cases had microduplication, seven cases had microdeletion, and one case had loss of heterozygosity (Table 4).

**TABLE 3:** The pathogenic CNVs in fetal CNS abnormalities.

Case	Microarray nomenclature	Size (Mb)	CNS abnormalities	Extra CNS abnormalities	Inheritance
1	arr [hg19]16p11.2 (29,567,296–30,190,029) × 1	0.6	Mild ventriculomegaly	-	*de novo*
2	arr [hg19]Xq28 (152,446,333–153,581,657) × 3,1p36.33p36.23 (849,466–592,172) × 1, 1q44 (246,015,892–249,224,684) × 3	1.1	Mild ventriculomegaly	-	*de novo*
		7.7			
		3.2			
3	arr [hg19] 7q36.3 (155,347,675–156,348,660) × 3	1.0	Hydrocephaly	-	*de novo*
4	arr [hg19]16p11.2 (29,591,326–30,176,508) × 1	0.6	Hydrocephaly	-	*de novo*
5	arr [hg19]16p11.2 (29,580,020–30,190,029) × 1	0.6	Spine malformation	-	*de novo*
6	arr [hg19]16p11.2 (28,819,028–29,051,191) × 1	0.2	Encephalocele	-	*de novo*
7	arr [hg19]17p12 (14,099,504–15,491,533) × 1	1.3	Posterior fossa widened	-	*de novo*
8	arr [hg19]22q11.2 (18,648,855–21,800,471) × 1	3.1	Choroid plexus cyst	-	*de novo*
9	arr [hg19]5q35.2q35.3 (175,416,095–177,482,506) × 1	2.0	Mild ventriculomegaly	Polyhydramnios	*de novo*
10	arr [hg19]15q11.2 (22,770,421–23,277,436) × 1	0.5	Dandy-Walker syndrome	VSD	Paternal
11	arr [hg19]16p13.3 (85,880–536,631) ×1, 17q24.2q25.3 (64,966,574–81,041,823) × 3	0.4	Mild ventriculomegaly	FGR, VSD, Persistent left superior vena cava	*de novo*
12	arr [hg19]17p13.3p13.2 (525–5,204,373) × 1	5.2	Mild ventriculomegaly, Cerebellar hypoplasia	Strephenopodia	*de novo*
13	arr [hg19]16p13.11 (15,422,960–16,508,123) × 1	1.0	Mild ventriculomegaly	Left ventricular hyperecho	*de novo*
14	arr [hg19]22q11.2 (20,730,143–21,800,471) × 1	1.0	Choroid plexus cyst	Strephenopodia, Polyhydramnios	*de novo*
15	arr [hg19]16p11.2 (28,810,324–29,032,280) × 1	0.2	Mild ventriculomegaly	Echogenic bowel, Left ventricular hyperecho	*de novo*
16	arr [hg19]15q13.2q13.3 (31,104,220–32,444,043) × 1	1.3	Holoprosencephaly (HPE)	CHD, Single umbilical artery	*de novo*
17	arr [hg19] 2q13 (111,397,196–113,111,856) × 1	1.7	Subependymal cyst	VSD, Persistent left superior vena cava, Thickened nuchal translucency	Maternal
18	arr [hg19]1p36.33p36.32 (849,466–4,894,800) × 1	4.0	Mild ventriculomegaly	Renal cysts	*de novo*

CNS, Central Nervous System; ACC, Agenesis of the corpus callosum; CNVs, copy number variations; CHD, congenital heart disease; FGR: fetal growth restriction; TP, termination of pregnancy; VSD: ventricular septal defect.

**TABLE 4:** The VUS CNVs in fetal CNS abnormalities.

Case	Microarray nomenclature	Size (Mb)	CNS abnormalities	Extra CNS abnormalities	Obstetrical outcomes	Inheritance
1	arr[hg19]1q21.1 (145,375,770–145,770,627) × 1	0.7	Mild ventriculomegaly	−	TD	−
2	arr[hg19] 16p13.11 (15,058,820–16,309,046) × 3	1.3	Mild ventriculomegaly	−	TD	Paternal
3	arr[hg19]3p22.1 (42,875,130–43,309,436) × 1	0.4	Mild ventriculomegaly	−	TD	−
4	arr[hg19]3p25.2 (12,183,082–12,669,247) × 3	0.5	Mild ventriculomegaly	−	TD	−
5	arr[hg19]3p26.3 (1,855,754–2,663,625) × 1	0.8	Mild ventriculomegaly	−	TD	−
6	arr[hg19]16q23.1 (75,275,963–76–432–398) × 3	1.2	Mild ventriculomegaly	−	TD	−
7	arr[hg19]3q26.1q29 (163,256,369–197,791,601)hmz,5p13.1p11 (41,029,137–46,313,469)hmz,6q24.2q25 (143,341,406–161,527,784)hmz,12q13.2q21.2 (56,011,100–77,134,151)hmz,17q21.2q21.32 (39,639,602–45,479,706)hmz,21q21.3q22.2 (28,124,165–42,352,287)hmz	99.1	Mild ventriculomegaly	−	TD	−
8	arr[hg19]14q21.2q21.3 (46,782,405–49,288,860) ×1	2.5	Hydrocephaly	−	TP	−
9	arr[hg19]15q13.3 (31,999,631–32,444,043) ×3	0.4	Hydrocephaly	−	TP	−
10	arr[hg19]2q36.1q36.2 (224,459,152–225,330,583) × 3	0.9	Posterior fossa widened	−	TD	−
11	arr[hg19]10q24.31q24.32 (102,972,457–103,179,063) × 3	0.2	Posterior fossa widened	−	TD	−
12	arr[hg19]18q21.33 (59,581,098–59,784,858) × 1	0.2	ACC	−	TD	−
13	arr[hg19] 5q35.3 (179,194,643–179,767,135) × 3	0.6	ACC	−	TD	Maternal
14	arr[hg19]20q13.2 (53,545,723–54,866,110) × 3	1.3	Choroid plexus cyst	−	TD	−
15	arr[hg19]15q13.3 (32,011,458–32,444,043) ×3	0.4	Choroid plexus cyst	−	TD	−
16	arr[hg19]8p23.2 (3,703,883–5,940,433) ×3	2.2	Choroid plexus cyst	−	TD	−
17	arr[hg19] 1q31.1 (186,148,297–190,257,668) × 3	4.1	Choroid plexus cyst	−	TD	Paternal
18	arr[hg19] 15q11.2 (22,770,421–23,625,785) × 1	0.8	Choroid plexus cyst	−	TD	Paternal
19	arr[hg19]1q21.1q21.2 (145,958,361–147,830,830) × 3	1.8	Subependymal cyst	CHD	TD	−
20	arr[hg19]11p15.1p14.3 (20,745,930–21,780,075) × 3	1.0	Mild ventriculomegaly	VSD, Hydronephrosis	TD	−
21	arr[hg19]18q11.2 (19,620,590–21,572,153) × 3	1.9	Mild ventriculomegaly	Tricuspid regurgitation	TD	Paternal
22	arr[hg19]15q13.3 (32,021,609–32,444,043) × 3	0.4	Mild ventriculomegaly	Echogenic bowel, Left ventricular hyperecho	TD	−
23	arr[hg19]1p31.3 (61,886, 890–63,701,576) × 1	1.8	Spine malformation	Thickened nuchal translucency	TP	*de novo*

CNS, Central Nervous System; ACC, Agenesis of the corpus callosum; CNVs, copy number variations; CHD, congenital heart disease; TD, term delivery; TP, termination of pregnancy; VSD: ventricular septal defect; VUS, uncertain clinical significance.

The rates of pathogenic CNVs were 2.5% (8/318) and 4.6% (10/217) in isolated CNS abnormalities and CNS abnormalities with other ultrasound abnormalities, respectively. The rate of pathogenic CNVs in CNS abnormalities with other ultrasound anomalies was higher than that in isolated CNS abnormalities, but the difference was not statistically significant (*P* = 0.187, *p* > 0.05) (Table 5).

**TABLE 5:** The rates of Pathogenic CNVs in isolated CNS abnormalities and CNS abnormalities with other ultrasound abnormalities.

Classification	Number of fetuses	Number of fetuses with abnormal CNVs	P CNVs	VUS CNVs	p-values
Isolated CNS abnormalities	318	26	8 (2.5%)	18 (5.7%)	−
CNS abnormalities with other ultrasound abnormalities	217	15	10 (4.6%)	5 (2.3%)	−
Total	535	41	18 (3.4%)	23 (4.5%)	0.187

p > 0.05 compared with isolated CNS anomalies and CNS abnormalities with other ultrasound abnormalities via χ^2^ test.

CNS, Central Nervous System; P, pathogenic; CNVs, copy number variations; VUS, uncertain clinical significance.

### The Detection Rate of Chromosomal Abnormalities and Pathogenic CNVs in Fetuses With Isolated CNS Abnormalities

The rate of chromosomal abnormalities and pathogenic CNVs in fetuses with isolated CNS abnormalities was 6.6%. The rates of chromosomal abnormalities and pathogenic CNVs in fetuses with spine malformation (50%), encephalocele (50%), subependymal cyst (20%), and microcephaly (16.7%) were higher than those with other isolated CNS abnormalities. The largest number of fetuses with isolated CNS abnormalities were 142 fetuses with mild ventricular enlargement, accounting for 44.7% (142/535) of the total, but the detection rate of chromosomal abnormalities and pathogenic CNVs was 2.8% (4/142) (Table 6).

**TABLE 6:** Phenotypic characteristics of 318 fetuses with isolated CNS abnormalities.

Isolated CNS abnormalities classifcation	Number of fetuses	Number of fetuses with chromosomal abnormalities	Number of fetuses with P CNVs (<10 Mb)	Number of fetuses with total anomaly
Mild ventricular enlargement	142	2	2	4 (2.8%)
Hydrocephaly	9	0	2	0
Posterior fossa widened	32	2	1	3 (9.4%)
ACC	13	1	0	1 (7.7%)
Cerebellar hypoplasia	4	0	0	0
Dandy-Walker syndrome	3	0	0	0
Spine malformation	4	1	1	2 (50%)
Encephalocele	2	0	1	1 (50%)
Microcephaly	6	1	0	1 (16.7%)
Choroid plexus cyst	89	5	1	6 (6.7%)
Arachnoid cyst	9	0	0	0
Subependymal cyst	5	1	0	1 (20%)
Total	318	12	9	6.6% (21/318)

ACC, Agenesis of the corpus callosum; CNS, Central Nervous System; CNVs, copy number variations; P, pathogenic.

### Obstetrical Outcomes

Among the 535 fetal CNS abnormalities, 523 were successfully followed up. The pregnancies were terminated for fetuses with chromosomal abnormalities (*n* = 36), pathogenic CNVs (*n* = 18), and VUS CNVs (*n* = 3). In addition, pregnancies were terminated for 25 fetuses with CNS abnormalities. Although karyotype analysis and SNP array showed normal results, 13 fetuses with severe nervous system malformations and 12 fetuses with severe multiple malformations accounted for the termination of respective pregnancies.

## Discussion

The main factor leading to the malformation of the central nervous system may be genetic. The malformation of the central nervous system may cause the brain ridge liquid circulation disorder, and left ventricle deformation or expansion. Central nervous system once appear abnormal, is likely to lead to fetal abnormal changes of several brain ventricles. Fetuses with CNS abnormalities have a poor prognosis with serious consequences, and due to their disabling nature, they pose a great burden to individuals, families, and society after birth. Therefore, it is necessary to take intervention measures such as intrauterine treatment and termination of pregnancy as early as possible.

In this study, SNP array and traditional karyotype analysis were performed on fetuses with CNS abnormalities with or without other ultrasound abnormalities. Traditional karyotype analysis detected 36 abnormal karyotypes, and the detection rate of chromosomal abnormalities was 6.7%. In addition to the 36 cases of CNVs consistent with karyotype analysis, SNP array detected 41 abnormal CNVs (an additional detection rate of 7.7%). These 41 abnormal CNVs were different in sizes and could not be identified by traditional karyotype analysis. The results of this study indicated that the etiologies of fetal CNS abnormalities were related to chromosomal microdeletions and/or microduplications, in addition to chromosomal abnormalities. The use of SNP array can make up for the shortcomings of the traditional karyotype analysis. For fetal CNS abnormalities with normal karyotype analysis, SNP array should be suggested for further detection.

Among the chromosomal abnormalities in fetuses with CNS abnormalities, trisomy 18 and chromosomal structural abnormalities were the most common, accounting for 31.4% (11/36) and 34.3% (12/36), respectively, followed by trisomy 21, trisomy 13, and other chromosomal number abnormalities. The detection rate of chromosomal abnormalities in CNS abnormalities with other ultrasound abnormalities was significantly higher than that in isolated CNS abnormalities (4.1 and 10.6%, respectively, *P* = 0.003). This indicates that the abnormality of aneuploidy and large fragment chromosome causes genetic changes and disturbs the balance between genes, thus leading to the malformation of fetal multi-systems (Hsiao et al., 2009). The rate of pathogenic CNVs in CNS anomalies with other ultrasound anomalies was higher than that in isolated CNS anomalies (2.5 and 4.6%), but the difference was not statistically significant (*p* > 0.05). Therefore, SNP array is recommended for fetuses with CNS abnormalities, especially for those with multiple CNS abnormalities. However, for fetuses with isolated CNS abnormalities, SNP array should not be considered because of their low incidence of microdeletions/microduplications.

The rates of chromosomal abnormalities and pathogenic CNVs in isolated CNS abnormalities was 6.6%. The rates of chromosomal abnormalities and pathogenic CNVs in fetuses with spine malformation, encephalocele, subependymal cyst, and microcephaly were higher than those with other isolated CNS abnormalities. Previous studies (Sun et al., 2015) have also demonstrated that isolated CNS abnormalities could still be detected at a rate of 6.5–8.3% in case of pathogenic CNVs by CMA, which could not be detected by traditional karyotype analysis.

Of the 41 cases with abnormal CNVs, 18 cases had pathogenic CNVs. Of these 18 pathogenic CNVs, eight cases were known as microdeletion/microduplication syndrome. There are many known pathogenic genes that can cause CNS abnormalities (Huang et al., 2012; Krutzke et al., 2016; Schumann et al., 2016). Eight cases with microdeletions were found in this study, including five cases with 16p11.2 microdeletion syndrome, two cases with 22q11.2 microdeletion syndrome, and one case with Miller Dieker syndrome (17p13.3p13.2 microdeletion). The most common is 16p11.2 microdeletion syndrome, with an incidence rate of 0.3% (Rosenfeld et al., 2010). The main clinical symptoms include intellectual disability and autism spectrum disorder (2015). The gene, T-box transcription factor 6, is a key gene causing vertebral deformity in patients with 16p11.2 microdeletion syndrome (Al-Kateb et al., 2014). At present, there are few reports on fetuses with 16p11.2 microdeletion syndrome. Only a few studies (Hernando, 2002; Li et al., 2017) have reported the ultrasound anomalies related to 16p11.2 microdeletion syndrome include cardiac malformation, polycystic kidney, absence of nasal bone, single umbilical artery, intrauterine growth retardation, etc. In this study, the five cases with 16p11.2 microdeletion syndrome had different ultrasound anomalies, including two cases with spine malformation, one case with hydrocephaly, one case with mild ventricular enlargement, and one case with other ultrasound abnormalities besides mild ventricular enlargement. SNP array showed that two cases had 22q11.2 microdeletion syndrome. The incidence of 22q11.2 microdeletion syndrome is about 1/4,000, and its clinical phenotypes are diverse (Sullivan, 2008). About 80% of cases with 22q11.2 microdeletion syndrome have various types of cardiac malformations (Ferencz et al., 1989). In this study, the two fetuses with 22q11.2 microdeletion syndrome showed no cardiac malformation. Among these two fetuses with 22q11.2 microdeletion syndrome, one showed only choroid plexus cyst, while the other one had choroid plexus cyst and varus and polyhydramnios. In this study, the SNP array of a fetus showed there was a 5.2 Mb deletion in p13.3p13.2 on chromosome 17, which could lead to the Miller-Dieker syndrome. The incidence of Miller-Dieker syndrome is about 1.2:100,000 (Kiiski et al., 2012; Roos et al., 2009), and is usually characterized by agenesis of the corpus callosum, microcephaly, etc (Tang et al., 2016). Ultrasound of this case in this study showed mild ventricular enlargement, cerebellar hypoplasia, and other ultrasound anomalies such as varus.

CMA also has its shortcomings, and cases with VUS CNVs make genetic counseling difficult. According to the literature (Breman et al., 2012; Faas et al., 2010; Hillman et al., 2011), the detection rate of VUS CNVs by CMA is 1.1–6%. In this study, among the 535 cases with abnormal CNS, 23 cases had VUS CNVs, and the detection rate of VUS CNVs was 4.3%, which was consistent with the previous reports. Among the 23 cases with VUS CNVs, eight cases were susceptibility loci for neurodevelopment, including three cases of 15q13.3 duplications, one case of 16p13.11 duplication, one case of 1q21.1q21.2 duplication, one case of 1q21.1 deletion, one case of 3p26.3 deletion, and one case of 15q11.2 deletion. The susceptibility loci for neurodevelopment had incomplete penetrance, and may be associated with cardiovascular disease, cognitive impairment, behavioral disorder, and intellectual disability (Idit Maya et al., 2018). The incomplete penetrance of the susceptibility loci for neurodevelopment poses a challenge for genetic counseling (Oneda and Rauch, 2017). Some VUS cases including 3p22.1 deletion, 3p25.2 duplication, 3p26.3 deletion, 14q21.2q21.3 deletion, 10q24.31q24.32 duplication, 5q35.3 duplication, 1q31.1 duplication, may only contain 1-3 OMIM genes, which is not related to the nervous system, but it was exceeds the reporting threshold. In case 12, 18q21.33 deletion contain the exon 16–31 of *PIGN*, homozygous mutations in this gene lead to multiple congenital abnormalities-hypokalemia-epilepsy syndrome, the clinical phenotype is lack of motor development, seizures, multiple malformations and various congenital abnormalities involving the heart, urinary and gastrointestinal systems. The fetal was a carriers of recessive genetic diseases caused by *PIGN* genes with no further testing. In recent years, as a new technology, next-generation sequencing has been used to detect single gene mutations and CNVs, and may provide a more comprehensive method to better assess the prognosis of the fetus.

Combining karyotype analysis with SNP array can help parents with fetuses having CNS anomalies to make decisions on whether to terminate pregnancy. For example, in fetuses with mild ventriculomegaly, normal results from SNP array indicate that the fetus may have a better prognosis, and parents can choose to continue pregnancy. In this study, among the 523 fetuses with CNS abnormalities that were successfully followed up, the pregnancies were terminated for 25 fetuses in which karyotype analysis and SNP array showed normal results. Thirteen of these cases had severe nervous system malformations and 12 cases had severe multiple malformations, which accounted for the termination of pregnancies. Therefore, even if the SNP array results were normal, most of the parents with fetal CNS abnormalities and multiple malformations still decided to terminate pregnancy, because of refractoriness to the postnatal treatment and seriousness in the postnatal phenotype such as severe deformity, intellectual disability, etc (Volpe and Joseph, 2010).

Comparing with the traditional karyotype analysis, SNP array can find more abnormal CNVs in fetuses with CNS abnormalities. It is suggested that SNP array should be used in the prenatal diagnosis of fetuses with CNS abnormalities, which can enable better prenatal assessment and genetic counseling, and also affect obstetrical outcomes.

## Data Availability

The original contributions presented in the study are included in the article/Supplementary Material, further inquiries can be directed to the corresponding authors.

## References

[B1] Hanson E. Bernier R. Porche K. Jackson F. I. Goin-Kochel R. P. Snyder L. G. (2015). The Cognitive and Behavioral Phenotype of the 16p11.2 Deletion in a Clinically Ascertained Population. Biol Psychiatry. 77, 785–793. 10.1016/j.biopsych.2014.04.021 25064419 PMC5410712

[B2] Committee Opinion (2016). Committee Opinion No. 682 Summary: Microarrays and Next-Generation Sequencing Technology: The Use of Advanced Genetic Diagnostic Tools in Obstetrics and Gynecology. Obstet Gynecol. 128, 1462, 1463. 10.1097/AOG.0000000000001814 27875471

[B3] Al-Kateb H. Khanna G. Filges I. Hauser N. Grange D. K. Shen J. (2014). Scoliosis and Vertebral Anomalies: Additional Abnormal Phenotypes Associated with Chromosome 16p11.2 Rearrangement. Am. J. Med. Genet. 164, 1118–1126. 10.1002/ajmg.a.36401 24458548

[B4] Breman A. Pursley A. N. Hixson P. Bi W. Ward P. Bacino C. A. (2012). Prenatal Chromosomal Microarray Analysis in a Diagnostic Laboratory; Experience with >1000 Cases and Review of the Literature. Prenat. Diagn. 32, 351–361. 10.1002/pd.3861 22467166

[B5] Cai M. Lin N. Su L. Wu X. Xie X. Li Y. (2020). Copy Number Variations Associated with Fetal Congenital kidney Malformations. Mol. Cytogenet. 13, 11. 10.1186/s13039-020-00481-7 32211073 PMC7092440

[B6] Cukier H. N. Dueker N. D. Slifer S. H. Lee J. M. Whitehead P. L. Lalanne E. (2014). Exome Sequencing of Extended Families with Autism Reveals Genes Shared across Neurodevelopmental and Neuropsychiatric Disorders. Mol. Autism. 5, 1. 10.1186/2040-2392-5-1 24410847 PMC3896704

[B7] Faas B. H. W. van der Burgt I. Kooper A. J. A. Pfundt R. Hehir-Kwa J. Y. Smits A. P. T. (2010). Identification of Clinically Significant, Submicroscopic Chromosome Alterations and UPD in Fetuses with Ultrasound Anomalies Using Genome-Wide 250k SNP Array Analysis. J. Med. Genet. 47, 586–594. 10.1136/jmg.2009.075853 20577003

[B8] Ferencz C. Neill C. A. Boughman J. A. Rubin J. D. Brenner J. I. Perry L. W. (1989). Congenital Cardiovascular Malformations Associated with Chromosome Abnormalities: an Epidemiologic Study. J. Pediatrics. 114, 79-86. 10.1016/s0022-3476(89)80605-5 2521249

[B9] Hadzagić-Catibusić F. Maksić H. Uzicanin S. Heljić S. Zubcević S. Merhemić Z. (2008). Congenital Malformations of the Central Nervous system: Clinical Approach. Bosn. J. Basic. Med. 8, 356–360. 10.17305/bjbms.2008.2897 PMC5677281 19125708

[B10] Hernando (2002). Comparative Genomic Hybridisation Shows a Partial De Novo Deletion 16p11.2 in a Neonate with Multiple Congenital Malformations. J. Med. Genet. 39, e24. 10.1136/jmg.39.5.e24 12011165 PMC1735111

[B11] Hillman S. C. Pretlove S. Coomarasamy A. McMullan D. J. Davison E. V. Maher E. R. (2011). Additional Information from Array Comparative Genomic Hybridization Technology Over Conventional Karyotyping in Prenatal Diagnosis: a Systematic Review and Meta-Analysis. Ultrasound. Obstet. Gynecol. 37, 6–14. 10.1002/uog.7754 20658510

[B12] Hsiao C.-C. Tsao L.-Y. Chen H.-N. Chiu H.-Y. Chang W.-C. (2009). Changing Clinical Presentations and Survival Pattern in Trisomy 18. Pediatrics. Neonatol. 50, 147–151. 10.1016/s1875-9572(09)60053-x 19750888

[B13] Huang J. Wah I. Y. M. Pooh R. K. Choy K. W. (2012). Molecular Genetics in Fetal Neurology. Semin. Fetal. Neonatal. Med. 17, 341–346. 10.1016/j.siny.2012.07.007 22909903

[B14] Maya I. Sharony R. Yacobson S. Kahana S. Yeshaya J. Tenne T. (2018). When Genotype is not Predictive of Phenotype: Implications for Genetic Counseling Based on 21,594 Chromosomal Microarray Analysis Examinations. Genet. Med. 20, 128–131. 10.1038/gim.2017.89 28726807

[B15] Kiiski K. Roovere T. Zordania R. Von Koskull H. Horelli-Kuitunen N. (2012). Prenatal Diagnosis of 17p13.1p13.3 Duplication. Case. Rep. Med. 2012, 840538. 10.1155/2012/840538 23118768 PMC3483775

[B16] Krutzke S. K. Engels H. Hofmann A. Schumann M. M. Cremer K. Zink A. M. (2016). Array-based Molecular Karyotyping in Fetal Brain Malformations: Identification of Novel Candidate Genes and Chromosomal Regions. Birth Defects Res A Clin Mol Teratol. 106, 16–26. 10.1002/bdra.23458 26680650

[B17] Li L. Huang L. Lin S. Luo Y. Fang Q. (2017). Discordant Phenotypes in Monozygotic Twins with 16p11.2 Microdeletions Including the SH2B1 Gene. Am J Med Genet 173, 2284–2288. 10.1002/ajmg.a.38284 28544142

[B18] Oneda B. Rauch A. (2017). Microarrays in Prenatal Diagnosis. Best. Prac. Res. Clini. Obstet. Gynaecol. 42, 53–63. 10.1016/j.bpobgyn.2017.01.003 28215395

[B19] Onkar D Onkar P Mitra K (2014). Evaluation of Fetal Central Nervous System Anomalies by Ultrasound and Its Anatomical Co-Relation. J Clin. Diagn. Res. 8, AC05–7. 10.7860/JCDR/2014/8052.4437 PMC4129269 25120962

[B20] Reddy U. M. Page G. P. Saade G. R. Silver R. M. Thorsten V. R. Parker C. B. (2012). Karyotype Versus Microarray Testing for Genetic Abnormalities after Stillbirth. N. Engl. J. Med. 367, 2185–2193. 10.1056/nejmoa1201569 23215556 PMC4295117

[B21] Roos L. Jonch A. E. Kjaergaard S. Taudorf K. Simonsen H. Hamborg-Petersen B. (2009). A New Microduplication Syndrome Encompassing the Region of the Miller-Dieker (17p13 deletion) Syndrome. J. Med. Genet. 46, 703–710. 10.1136/jmg.2008.065094 19520700

[B22] Rosenfeld J. A. Coppinger J. Bejjani B. A. Girirajan S. Eichler E. E. Shaffer L. G. (2010). Speech Delays and Behavioral Problems are the Predominant Features in Individuals with Developmental Delays and 16p11.2 Microdeletions and Microduplications. J. Neurodevelop. Disord. 2, 26–38. 10.1007/s11689-009-9037-4 PMC3125720 21731881

[B23] Sahoo T. Dzidic N. Strecker M. N. Commander S. Travis M. K. Doherty C. (2017). Comprehensive Genetic Analysis of Pregnancy Loss by Chromosomal Microarrays: Outcomes, Benefits, and Challenges. Genet. Med. 19, 83–89. 10.1038/gim.2016.69 27337029

[B24] Schumann M. Hofmann A. Krutzke S.K. Hilger A.C. Marsch F. Stienen D. (2016). Array-Based Molecular Karyotyping in Fetuses with Isolated Brain Malformations Identifies Disease-Causing CNVs. J. Neurodev. Disord. 8, 11. 10.1186/s11689-016-9144-y 27087860 PMC4832534

[B25] Sullivan K. E. (2008). Chromosome 22q11.2 Deletion Syndrome: DiGeorge Syndrome/Velocardiofacial Syndrome. Immunol. Allergy. Clin. North. Am. 28, 353–366. 10.1016/j.iac.2008.01.003 18424337

[B26] Sun L. Wu Q. Jiang S.-W. Yan Y. Wang X. Zhang J. (2015). Prenatal Diagnosis of Central Nervous System Anomalies by High-Resolution Chromosomal Microarray Analysis. BioMed. Res. Int. 2015, 1–9. 10.1155/2015/426379 PMC4443641 26064910

[B27] Tang XH Yang BC Zhu S Su J Zhang JM Yin YF (2016). Prenatal Diagnosis of Chromosome Abnormalities and Nine Nicrodeletion Syndromes using both Traditional Karyotyping and BoBs. Zhonghua fu chan ke za zhi 51, 325–30. 10.3760/cma.j.issn.0529-567X.2016.05.002 27256438

[B28] Volpe Joseph J (2010). Neurology of the Newborn. Major Probl Clin Pediatr. 1, 22. 10.1111/j.1469-8749.1959.tb08069.x 7022034

